# Exploring the perceptions of medical students about the effects of financial stresses in Pakistan

**DOI:** 10.12669/pjms.41.2.9286

**Published:** 2025-02

**Authors:** Nowshad Asim, Brekhna Jamil, Ayaz Ul Haq, Afreenish Malik

**Affiliations:** 1 Dr. Nowshad Asim, MBBS, DFM, MHPE. Assistant Professor, Institute of Health Professions Education and Research (IHPER), Khyber Medical University, Peshawar, Pakistan; 2 Dr. Brekhna Jamil, BDS, MPH, MHPE, PhD. Professor & Director, Institute of Health Professions Education and Research (IHPER), Khyber Medical University, Peshawar, Pakistan; 3 Mr. Ayaz Ul Haq, Institute of Health Professions Education and Research (IHPER), Khyber Medical University, Peshawar, Pakistan; 4 Dr. Afreenish Malik, BDS, MHPE Lecturer, Institute of Health Professions Education and Research (IHPER), Khyber Medical University, Peshawar, Pakistan

**Keywords:** Academic performance, Career choices, Debt, Financial stress, Medical students

## Abstract

**Objective:**

To explore the perceptions of medical students about the effects of financial stresses.

**Method:**

A qualitative exploratory study was conducted in March – August of 2023. Data were taken from a purposive sample of twelve undergraduate medical students from various medical colleges in Peshawar. Semi-structured in-depth interviews were conducted using a validated interview guide to explore the effects of financial stress on medical students. The audio recordings of the interviews were transcribed. Because the data was sensitive, anonymity and confidentiality were guaranteed to the participants. Thematic analysis was then used and authors of the study agreed on themes and subthemes after a manual thematic analysis was completed.

**Results:**

This study reveals five themes about the effects of financial stress among medical students. The five themes include financial stressors in medical education, their effects on academic performance and achievement, future career choices, healthcare and well-being practices, and educational policies and interventions suggesting potential solutions to overcome the financial challenges.

**Conclusion:**

This study highlights the multifaceted nature of financial stress among medical students, its significant influence on academic achievement and career decisions, and the negative health implications it presents. It also explores potential strategies for alleviating this burden. In addition to the suggestions given by medical students, there is a need to adopt specific strategies for medical institutions, policymakers, and future research endeavors to deal with the financial challenges faced by medical students.

## INTRODUCTION

The high cost of medical education has grown to be a major issue, with significant effects on students’ health and academic achievement.^1^,^2^ The escalating expenses associated with medical education have garnered significant scholarly and public interest in recent decades.^3^ For example, the median medical school cost of attendance in the US exceeded $275,000 in 2020, indicating a critical problem that affects a large segment of this student population.^4^ Students with financial difficulties usually get personal loans, bank loans, or educational debts to cover these costs.^5^ Stress related to money has been connected to several detrimental health effects, such as sleep difficulties, anxiety, depression, and effects on educational achievement.^5^-^8^ Exorbitant loans and debts may impact the career choice of medical students causing a push towards highly paid specialties.^7^ Such debts and financial stresses negatively affect the well-being, academic performance, and influence the career choice of students.^3^,^9^

Although research conducted by Iqbal et al. (2022) on 259 medical students in Pakistan has concentrated on quantifying the association of financial stress, student’s positivity, their academic achievement and also examining the mediating role of family conflicts,^10^ this study offers an exploration of the perceptions and lived experiences of medical students about the adverse effects of financial difficulties.^11^ The study intends to enhance the current scholarly conversation by providing a more nuanced understanding of the problem. It is of vital importance to highlight the need for reforms in the financing of medical education and the provision of support for medical students who experience financial stresses.^12^

By examining the experiences and perspectives of medical students, this study aims to fill a vacuum in the literature and contribute to customized interventions that reduce financial constraints and improve overall well-being. This study, which aimed to explore the consequences of financial stress on medical students’ mental health, academic achievement, and career choices, is significant in the Pakistani setting. It provides evidence for developing focused policies that support good financial stress management.

## METHODS

A qualitative exploratory study was conducted during March – August of 2023. Data were taken from a purposive sample of twelve final years medical students from medical colleges located in Peshawar. After Advanced Studies and Research Board (AS&RB) approval at Khyber Medical University in May 2023 (No: DIR/KMU-AS&RB/EP/002026 Dated: June 12, 2023)

### Ethical Approval:

It was taken from the KMU-IHPER Ethical Board (Ref No:1-11/IHPER/MHPE/KMU/23-32 Dated: June 13, 2023).

### Data collection:

For data collection, an interview guide with open-ended validated questions was used to explore the medical students’ perspectives about the effects of financial stresses. The interview guide was under the supervisor’s guidance. The guiding questions were searched through literature under the supervisor’s guidance which were subsequently authenticated by experts in the field of medical education. The questions in the interview guide were piloted with two final-year students. Before starting the interview, the interviewer assured the participant about the confidentiality. An interview guide was used to conduct in-depth interviews with twelve participants after informed consent. The interviewer opened the discussion by posing questions, guiding the respondent, using appropriate body language, and communicating the purpose of the study and its implications. All the interviews were audiotaped and notes were made. Key points were summarized at the end of the interview. Each participant met with the same interviewer.

All interviews were audio recorded and transcribed accurately. Because the data was sensitive, all the responses were kept anonymous and strictly confidential. The anonymity and confidentiality were confirmed to the study participants before the interviews. After that, the transcripts were made anonymous before discussing with co-authors for data analysis. Thematic analysis was then used and all authors agreed on themes and subthemes after a manual thematic analysis was completed.

### Data analysis:

Discussion points were transcribed from audio recordings. Analysis of the text was done using an open coding technique. The interviewer, three medical educationists from the health professions education reviewed the transcripts after each session and coded that data under several headings. Each transcript was returned to each participant for comments and corrections if required. The codes and themes were again shared with the three health professions educationists to sort out any possible differences and subsequently, agree upon by consensus. Any point which needed clarity was used as a guide for the next session. Themes emerged from this process, which were then reviewed by the co-authors till a consensus was reached on themes. Participant’s quotations were presented to illustrate themes with the referring number of each participant. The themes that emerged were ensured for their clarity. Both data collection and data analysis were done simultaneously. The final report was written and was then submitted to the correspondence author for approval. With the aim of pragmatism, a fresh interpretation of the data was done and any preconceived theoretical frameworks were not included. In this study, data triangulation was incorporated to enhance the credibility and reliability of findings through interviews and observations of participants from different medical colleges. The process involved member checking of transcripts, ensuring that participants confirmed the accuracy of their responses, and included reviews by supervisors and medical educationists to achieve consensus on themes. The transferability of the findings is supported by the study’s diverse sample, which spans both public and private sector institutions.

## RESULTS

All the study participants were final-year medical students, selected through purposive sampling identified through the Social Welfare Society of the colleges, as students who were unable to self-support or sustain themselves financially and had taken loans. This selection ensured reflected students facing significant financial challenges, which is central to the study’s objectives. Out of 12 participants, eight were males (66.7%) and four were females (33.3%). A total of five themes with eighteen subthemes emerged after the data analysis (Table-I).

**Table-I:** Perceptions of medical students about the effects of financial stresses.

	Participant’s quotations
***Theme 1: Financial stressors in medical education*** Sub themes Psychosocial Impact of Financial Strain Navigating Affordable Living Student Loan Management and Financial Literacy Limited opportunities of scholarships and financial aid programs	*“I would like to say that financial stress is one of the biggest challenges for a medical student.”* ***(M, R***#**5)** *“To be honest, we all know that medical school is quite hectic on its own. But when it comes to financial burdens, it puts a lot of stress on the student.”* ***(M, R#7)*** *“Financial stress can be in the form of money deficiency, or the needs of a medical student which could not be met due to shortage of money.”* ***(F, R#4)*** “I find that financial stress is the failure in your day-to-day needs which are events happening in college and living in the hostel.” ***(M, R#2)*** “The most important and difficult to manage are my daily expenses particularly hostel mess charges, canteen charges at college, and the yearly college tuition fees give me a tough time.” ***(F, R#9)*** “I think the financial stress is all due to the inability to pay the medical college dues and all other related expenditures of the five-year study period out there including every experience that can be dependent on money.” ***(M, R#11)*** “I perceive financial stress as a source of huge financial burden on students and their parents. I eat less, don’t spend on irrelevant things and as a medical student usually borrow all sorts of things from roommates or classmates like books, stationary etc” ***(M, R#12)***
***Theme-2 Academic Performance and Achievement subthemes*** Financial barriers to education Psychologic consequences of financial stresses on academic performance Poor academic performance as a consequence of financial challenges	*“There is a huge impact on the life of students, because if we cannot manage the tuition fees and hostel dues as well as home situations, how can we progress in studies?”* ***(M, R#1)*** “My academic performance has been badly affected by such thoughts and fear of being short of money. My grades have been gradually going down. I failed in one or two subjects in annual examinations, and then I had to pass those papers in the supplementary exam.” ***(F, R#4)*** *“Financial stresses have affected my academic performance during annual and supplementary exams. I have been struggling to focus on my studies due to financial worries which has resulted in my poor grades and I usually score very low marks as compared to the efforts I put into my studies.”* ***(F, R#9)***
***Theme-3 Future Career Choices*** subthemes Career paths restricted by financial challenges Financial responsibility and supporting the family Identifying Career Paths Aligned with Financial Needs Career development stages and strategies	*“It has very much affected my future choices. To focus on long courses and specializations needs much time and tolerance, but now I am in such a condition that I cannot afford or don’t have the time to pursue those long careers.”* ***(M, R#2)*** “It was my mother’s dream and I also wanted to become a gynecologist. But when I look into my financial problems, I think that due to these financial burdens, I cannot achieve my dream because of that.” ***(F, R#6)*** “This financial stress has influenced my future planning a lot like I want to earn more money so that I can support my family and my siblings so they do not have to go through same troubles and problems.” ***(F, R#4)*** *I have limited options so I will have to start my career as soon as possible.”* ***(M, R#2)*** “My future plans are getting affected as I will choose the field which is best to deal with my financial problems. Nowadays, as I’m thinking of orthopaedic and neurology or cardiology, about these three fields, I am thinking these fields can help me in future.” ***(M, R***#**5)** “I would go for surgical specialties if I could but actually, I want to earn money as soon as I complete MBBS to compensate for all my educational expenses which are in millions of rupees.” ***(M, R#10)*** “I want to do postgraduation in specialties which would pay off my debts as well as improve my financial status so that my father and family are relieved of the financial burdens, they have been going through for so long just because of my MBBS study.” ***(M, R#11)*** “I would go for the high-paid specialty and want to compensate for all my previous financial deprivations. For this, I have decided on Cardiology to peruse after my MBBS completion.” ***(F, R#9)***
***Theme-4 Healthcare and Well-being Practices*** subthemes Health-related decisions influenced by financial stresses Enhancing psychiatric counselling support for students	“In my daily life, I was going under depression and then I got a permanent depression. It very much affected my daily life.” ***(M, R#1)*** “I had good grades in my school years. I always got A-plus grades in my school, but until the financial crisis came in over my life, then I became very much depressed, and now that I get low grades.” ***(M, R#2)*** “Actually, I am having panic attacks and a lot of depression. I’m going through anxiety. These symptoms of depression are affecting my health. I have started losing weight.” ***(F, R#6)*** *Financial stresses have influenced my life both physically and mentally. I am doing very hard work daily including going on to classes, and then going to tuition services, etc. I am doing tuition as part time job due to financial stress. I don’t have proper time for myself, it is physically affecting me. And mentally, it has affected me a lot. I can’t focus on anything. My concentration is being diverged. There is a diversion in my concentration, I can’t concentrate on my studies, on my goals, my family and on my activities, which are important.* ***(M, R#5)*** “I’ve become a very aggressive person because of that, I don’t know why. I usually shout at my siblings and others at home. When I’m not financially stable, I usually yell out at people; I usually keep myself occupied in the corner of my room.” (M, R#7)

*M: Male, F: Female, R: Respondent.

## DISCUSSION

The study has highlighted the perspectives and lived experiences of medical students about the effects of financial stresses during student life in medical colleges. It aimed at comprehensively understanding financial stress among medical students, and found clear resonance in findings. The theme “Financial Stressors in Medical Education” unearthed several factors contributing to this stressful situation. This stress encompasses tuition fees, daily financial pressures, and more, significantly influencing the daily lives of medical students. Moving on, our investigation examined the impact of financial stress on academic achievement, identified by the theme “Academic Performance and Achievement.” It revealed a strong connection between financial stress and academic hurdles. Students’ quotations portrayed the challenges they face in maintaining focus on their studies, leading to diminished academic performance, including lower grades and occasional exam failures. This study explored the influence of financial stress on students’ future career choices, as revealed through the theme “Future Career Choices.” This theme highlighted how financial difficulties significantly impact career choices. Many students opt for higher-paying, shorter specialty paths to mitigate their financial burdens, diverting from their initial career aspirations. The effects of financial stress on healthcare practices and overall well-being, are revealed by the theme “Healthcare and Well-being Practices.” It shed light on the prevalence of anxiety, depression, and panic attacks among students contending with financial stress. These mental health challenges transcended academics, manifesting physically in issues like weight loss and diet difficulties. Identifying potential solutions, found its expression in the fifth theme “Educational Policies and Interventions.” Here, participants offered recommendations, including broadening scholarship programs, implementing soft loan initiatives, and enhancing financial counselling services. This theme underscored the capacity of institutions and policymakers to make meaningful changes in medical students’ lives.

The safety needs within Maslow’s hierarchy, also include financial security, and health and well-being are more fundamental.^13^ The issue of financial stress among medical students requires the attention of policymakers as well as educational institutions to establish support structures that may effectively mitigate this burden.^14^ To reduce the impact of financial stress on students, it is recommended to use several strategies such as facilitating access to scholarships, offering financial counselling services, and organizing seminars focused on financial management.^15^ These interventions aim to provide medical students with the necessary skills and awareness to properly handle their financial responsibilities throughout their educational journey in medical schools.^16^ Students also described financial stresses limiting their future choices such as limiting opportunities to pursue medical mission work or to work part-time. Students experienced these limitations negatively. Governmental interventions through a limit on tuition fees and capping a significant percentage of seats in private medical colleges can sort this issue.^14^ The suggestion to introduce scholarships for meritorious and financially weak students can be initiated or prompted by the accreditation bodies within private medical institutions.^17^,^18^ In future research, exploration and quantification of the relationships between medical students ‘debts, their debt or loan amount, and their career intentions are needed in medical colleges of Pakistan.^11^

Our analysis of these results demonstrates a strong alignment with the research objective. These findings offer a nuanced comprehension of the pervasive financial stress among medical students, affecting multiple aspects of their lives.

### Strengths:

The study explored the perceptions of medical students studying in medical colleges about the effects of financial stress and challenges affecting their lives, health, academic progression, and future career choices. Based on their lived experiences, the participants of the study provided several recommendations that could be potentially beneficial in lessening their financial challenges.

### Limitations:

The study is limited to medical colleges only. It is suggested to include other health sciences institutions for future research to bridge this gap to get a broader spectrum of students’ perspectives for a more holistic understanding of the subject matter. Moreover, further exploration and quantification of the relationships between students ‘debts, their debt or loan amount, and the career actually chosen are needed in medical colleges of Pakistan.

## CONCLUSIONS

The findings of the study have applications for Pakistan’s higher education establishments. Institutions can improve the effectiveness of financial aid programs and mental health support services by exploring the nature of financial issues that medical students face. This understanding may also result in the changes to academic policies that alleviate the negative impact of financial hardships on academic performance. The study may have an impact on Pakistani national policy in addition to institutional impact. By emphasizing the necessity of financial support for students facing financial strain and changes in medical education funding, the study could help ensure a steady stream of medical professionals enter the country’s healthcare system.

Finally, this study lays the groundwork for future investigations into the relationship among medical students between financial strains, academic success, psychological health, and career opportunities. By clarifying the interdependencies between these variables, the study opens the door to more thorough and integrative approaches to tackling these issues in subsequent research projects.

### Authors’ Contribution:

**NA BJ:** Conception, Design, literature search

**AUH:** Data analysis, interpretation, critical review

**AM:** Critical review, editing, literature search

All authors have read the final version and are accountable for integrity of the study.
